# Patient, informal caregiver and care provider acceptance of a hospital in the home program in Ontario, Canada

**DOI:** 10.1186/1472-6963-7-130

**Published:** 2007-08-17

**Authors:** Jacques Lemelin, William E Hogg, Simone Dahrouge, Catherine Deri Armstrong, Wei Zhang, Jo-Anne Dusseault, Joy Parsons-Nicota, Raphael Saginur, Gary Viner

**Affiliations:** 1 C.T. Lamont Primary Health Care Research Centre, Élisabeth Bruyère Research Institute, Rue Bruyère St., Ottawa, Canada; 2 Civic Family Medical Centre, University of Ottawa, Ottawa, Canada; 3 Department of Economics, University of Ottawa, Ottawa, Canada; 4 Riverside Family Health Team, University of Ottawa, Ottawa, Canada; 5 Department of Medicine, Ottawa Hospital, Ottawa, Canada

## Abstract

**Background:**

Hospital in the home programs have been implemented in several countries and have been shown to be safe substitutions (alternatives) to in-patient hospitalization. These programs may offer a solution to the increasing demands made on tertiary care facilities and to surge capacity. We investigated the acceptance of this type of care provision with nurse practitioners as the designated principal home care providers in a family medicine program in a large Canadian urban setting.

**Methods:**

Patients requiring hospitalization to the family medicine service ward, for any diagnosis, who met selection criteria, were invited to enter the hospital in the home program as an alternative to admission. Participants in the hospital in the home program, their caregivers, and the physicians responsible for their care were surveyed about their perceptions of the program. Nurse practitioners, who provided care, were surveyed and interviewed.

**Results:**

Ten percent (104) of admissions to the ward were screened, and 37 patients participated in 44 home hospital admissions. Twenty nine patient, 17 caregiver and 38 provider surveys were completed. Most patients (88%–100%) and caregivers (92%–100%) reported high satisfaction levels with various aspects of health service delivery. However, a significant proportion in both groups stated that they would select to be treated in-hospital should the need arise again. This was usually due to fears about the safety of the program. Physicians (98%–100%) and nurse practitioners also rated the program highly. The program had virtually no negative impact on the physician workload. However nurse practitioners felt that the program did not utilize their full expertise.

**Conclusion:**

Provision of hospital level care in the home is well received by patients, their caregivers and health care providers. As a new program, investment in patient education about program safety may be necessary to ensure its long term success. A small proportion of hospital admissions were screened for this program. Appropriate dissemination of program information to family physicians should help buy-in and participation. Nurse practitioners' skills may not be optimally utilized in this setting.

## Background

Within Canada, current policy priorities include enhancements to primary healthcare and home care [1]. The role of the nurse practitioner is being developed and expanded as an important workforce augmentation [1]. The consensus is for regions to decentralize care, moving services that are traditionally performed in acute hospitals, out to the community. Rationales for this move include patient independence, generally lower costs and improved quality of life. Expanding the scope of health care delivery within the home, such as with "Hospital in the Home" (HITH) or "Hospital at Home" models [2,3], offers the potential to address these trends with an expanded role for Family Medicine [4].

Basic requirements of these models are the provision of care in the patient's home that: (1) reduces or eliminates inpatient hospital stay; (2) is similar to care provided in hospital and clinically appropriate; and (3) is not provided by usual community based home care services. HITH programs may allow for the re-allocation of beds to deal with wait times, and the provision of culturally appropriate care, while reducing iatrogenic complications, and public costs [5].

An evaluation of such programs should have multiple dimensions. The program must achieve quality patient outcomes, be economically efficient, and be acceptable to those involved [6,7]. A successful program should meet the needs and expectations of patient and informal caregivers (family and/or friends), and entail job demands that are acceptable to professional healthcare providers. Most studies evaluating stakeholder acceptance have focused on the perspectives of patients and their caregivers. Few studies have examined the perspectives of healthcare providers [8-10]. Understanding the level of acceptance from all parties is essential in determining the success of HITH programs.

HITH programs have been successfully implemented in several countries and may be one approach for the Canadian healthcare system to address its policy priorities. However, HITH interventions may have different components and varied outcome potentials in different health care systems. The current study evaluates the acceptance of a HITH program, managed and provided by nurse practitioners in a family medicine program, among patients, caregivers, nurse practitioners, and physicians (hospital staff residents, attending physicians, and family physicians) in one community in Ontario, Canada.

## Methods

### Design

We used surveys and semi-structured interviews to evaluate patient, caregiver and care provider acceptance of a new HITH intervention.

### Location

The study was conducted in the province of Ontario, in Canada. Canada has a public funded health care system which covers 100% of the hospital services costs.

### Participants

**Figure 1:**
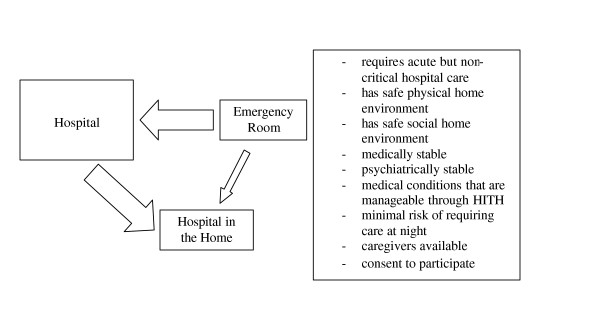
**Eligibility**. Inclusion criteria and recruitment of patient participants.

Participants were recruited from the 14 bed inpatient unit of the Family Medicine Service (FMS) or directly from the emergency department of the Ottawa Hospital, Civic Campus if they required admission to the FMS (Figure 1). Participants had to: require acute but non-critical hospital care; have safe physical and social home environments; be medically and psychiatrically stable; have medical conditions that were manageable within the service limits of the HITH program and present minimal risk of needing care at night, have informal caregivers or caregiver networks available. They were required to give consent to participate. The Ottawa Hospital Research Ethics Board approved this study.

### Care provision

Patient management remained the principal responsibility of the hospital attending physician. However, Nurse practitioners (NPs) were the designated principal home care provider. The lead program NP was seconded from department of family medicine for the period of the study. Additional NPs were hired to the FMS and trained for their role as home care provider in the HITH study. Direct care was delivered in patients' homes almost exclusively by NPs, who provided rehabilitative and supportive care, including education, coordination of services, and counseling. In Ontario, NPs are licensed as extended class Registered Nurses. The NPs worked for nine hours daily from Monday to Friday, and four hours daily on weekends. The FMS family physicians call group provided after hour coverage for patients as well as backup support to NPs. Immediately following each patient's transfer to the home, an NP made a home visit, performed a physical examination and initiated care provision. The NP continued to visit the patient daily and maintained telephone contact until discharge. The provision of care was based on the patient's care needs and was determined in consultation with the family medicine resident, attending staff physician and other health care professionals at the time of transfer to the home. Care was reviewed with the hospital medical team as required, throughout the care period, and patients were discharged from the HITH service when the team agreed there was no further requirement for hospitalization.

### Data collection

Upon admission, the NPs recorded patient demographic information, such as the patient's living arrangement and current main activity. NPs also administered both the Short Form-12 (SF-12) quality of life questionnaire (4 weeks version) [11] and Health Related Quality of Life-4 (HRQOL-4), which captures the patient's perceived health status and number of unhealthy days in the past 30 [11,12].

Program acceptance was evaluated using a questionnaire adapted from another Ontario HITH study [13]. At discharge, a research associate contacted each patient and their informal caregivers, by telephone, to administer a survey relating to their satisfaction and level of acceptance with the program. Caregivers were also asked about any additional burden brought on by the program. A question referring to services outside the scope of the intervention was included in the patient questionnaire to act as a point of reference for satisfaction ratings. The tools used were adapted from other surveys for the purpose of this study.

An assessment survey was mailed, immediately following discharge, to the patient's family physician and the hospital medical staff (resident and attending) overseeing the care of the patient. Questions pertained to their perception of the quality of care delivered, the quality of the collaboration with the nurse practitioner and the impact of the program on their practice.

NPs involved in the program completed a survey and were interviewed with questions addressing program suitability and job satisfaction midway through the 18 month study period. Interviews were coded by hand to identify thematic areas and triangulate with the quantitative data.

On all surveys, satisfaction questions were assessed on a 5 point Likert scale. Other questions required yes/no answers or short written responses.

### Statistics

Sample size calculation was based on quality of care outcomes. Based on a previous randomized controlled trial [2], we estimated that 50 patients would be required to have adequate study power (80%) (manuscript in preparation). In this study we report mainly descriptive statistics. The sample size was not sufficiently large to allow for sub-group analyses.

## Results

### Eligibility

During an 18 month period (November 2003–May 2005), approximately 1,000 patients were admitted to the FMS ward and received in-hospital care for an average of 8.4 days. Of these, 104 admissions were closely screened for participation in the study. The majority of patients were not considered because of obvious non-eligibility, usually evidently too well or too ill. Also, while the NP had home admissions to manage, the time spent reviewing potentially new patients was limited. Of those screened, twelve patients refused participation in the program, half of whom felt they were too sick to go home. Forty eight were deemed ineligible in accordance with the exclusion criteria (11 did not require acute care/hospital admission, 20 were not medically or psychiatrically stable, 6 did not have a caregiver available, 3 lived too far away and 8 were not patients of the clinical practice involved in the study). Thirty seven eligible patients agreed to be enrolled in the program, representing 44 different admissions to the program. Three patients had two, and two patients had three admissions into the program (each admission requiring a separate consent). Among the 44 admissions, 4 were admitted directly from the emergency room and 40 were transferred from the inpatient hospital ward, after an average 6.3 days stay, to the HITH program. In each case, the patient would have required a continued in-hospital stay.

### Patient profile

Patients were usually older adults, living with relatives or friends and not working. Demographic information is presented in Table 1. Some (38%) of participants were also recipients of community care services at the time of their admission. Details of these services were not available. The most common diagnoses were chronic obstructive pulmonary disease (exacerbation, or lower respiratory tract infection) (32%), cellulitis (11%), diabetes (uncontrolled or ketoacidosis) (9%) and congestive heart failure (9%). Other diagnoses included: nephritis, alcoholic cirrhosis of the liver, duodenal ulcer with hemorrhage, suspected C difficile enterocolitis, epilepsy, skull fracture, and pneumonia. At the time of admission into the program, 56% of patients expressed that, in general, they perceived their health as fair or poor. The mean number of self-reported "unhealthy days" in past 30 days was 19. SF-12 physical (31, 95% CI: 28–34) and mental (42, 95% CI: 38–46) health summary scores were indicative of a population with poor physical health and moderate deficiencies in mental health.

**Table 1:** Patient demographics (n = 37)

**Age (years) – median (range)**	75 (25–91)	
	**n**	**%**

**Sex (male)**	18	49
**First language**		
English	30	81
French	2	5
Other	5	14
**Living arrangement**		
Live alone	13	35
Live with relative or friend	24	65
**Receiving community care services**	14	38
**Highest level of education (n = 35)**		
<= high school	22	63
Community college or University	13	37
**Current main activity**		
Working for pay or profit	5	14
Recovering from illness/on disability	5	14
Caring for family, retired, other	27	73
**Total income (n = 33)**		
< $20,000	8	24
$20,000–$39,999	13	39
$40,000–$59,000	8	24
>$60,000	4	12

### Patients' perspective

Twenty nine patient surveys were completed. One patient with three admissions and one with two admissions responded to the first survey but not subsequent ones, five patients refused, five others could not be contacted, and two died within weeks after discharge of unrelated events. Too few patients were enrolled to allow a detailed comparison between responders and non-responders, except to suggest that non-respondents were more likely to be working for pay (25% vs 8%) but were otherwise similar to respondents.

**Figure 2:**
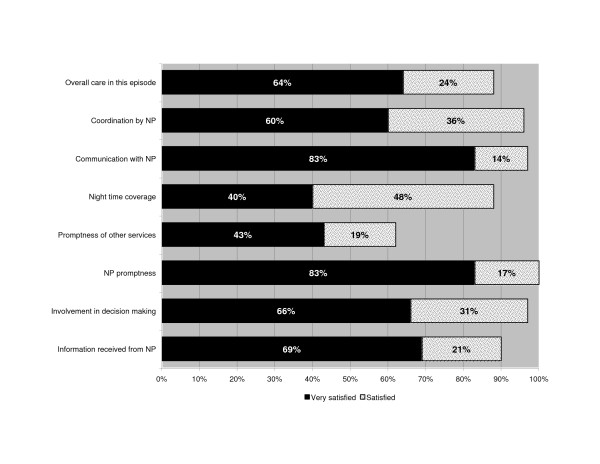
**Results of patient survey**. Patient satisfaction with services.

The majority reported high levels of satisfaction across all survey questions regarding the HITH program (range of satisfied/very satisfied): 88% – 100%. In contrast, the reference question measuring satisfaction with the promptness of other services had moderate satisfaction levels (satisfied/very satisfied 62%) (Figure 2). Most (79%) reported having learned how to better manage their illness. This is especially significant since many (48%) had suffered from the same condition in the past year, the majority of whom (83%) had required earlier hospitalizations for these conditions.

Most (83%), felt "confident" or "very confident" that the program was right for them. However, when asked to identify their preferred site (hospital or home) for their care, 37% reported a preference for hospital. Reasons given included "*knowing that treatment would be steps away*", and that it was "*scary being left alone at home*". Some selected "hospital" and stated that "*If in very bad shape ... then [I would rather be in] hospital but [I would] prefer home if [I] can manage*". The concern was usually around the evening and night period when the NP was not available. All patients had been safely managed without significant adverse events or mortality. Two patients were discharged from the HITH to be re-admitted to the in-patient service on the same day or following day. This was for the management of a lower respiratory tract infection in a patient with COPD, and pneumonia in another with diabetes. One of these patients selected hospital as the preferred site of hospitalization, and the other did not voice a preference. There was no apparent relationship between the selection of site and the overall satisfaction level (satisfied and very satisfied), education level, sex, and distribution of diagnoses or living arrangement. However, patients with previous hospitalization (70% vs 34%) for the same illness and those with more complex admissions (60%, 44%, and 25% for complexity level 3–4, 2, and 1, respectively) appeared more likely to select hospital, but this did not reach statistical significance.

### Informal caregivers' perspective

Seventeen caregiver surveys were completed. The caregivers of patients with multiple admissions completed a survey for the patients' first admission only; 13 refused, and 7 others could not be reached. We had not collected caregiver information on non-respondents that would allow a comparison with respondents.

The caregivers were mostly females (82%) and the majority were spouses of the patients (53%). Their average age was 66 (95% CI: 59–72). Few (18%) had remunerated work, and all who did, missed work during the home hospitalization period. When asked to put a dollar value on their role in the care of their family member in the program, the majority did not expect compensation for this type of work: four said $10–15/hour and one would expect payment for lost wages. The added costs associated with the program were identified as an increased amount of laundry (3), food (2), electricity (required for an oxygen machine, 1), and the purchase of a new bed (1).

**Figure 3:**
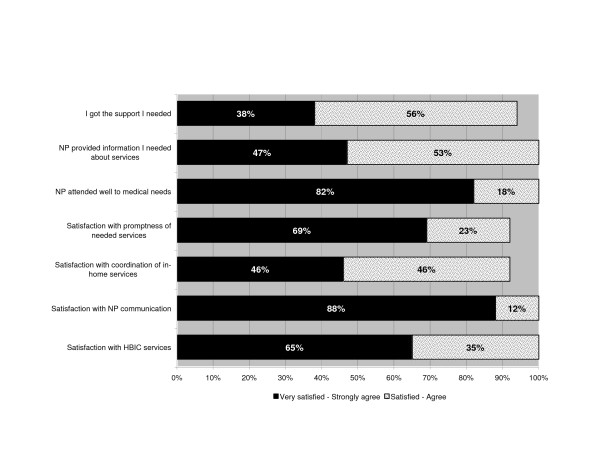
**Results of caregiver survey**. Caregiver satisfaction with services.

The majority (77%), felt prepared for what to expect during the home hospitalization, and almost all (94%) said they had been comfortable managing the patient in the home. The feedback provided on various aspects of health service delivery was positive ("satisfied"/"very satisfied" or "agree"/"strongly agree") (range 92% – 100%) (Figure 3).

Informal caregivers were also asked to identify whether the hospital or home was the preferred location of care for this episode. A considerable minority preferred hospital (35%). This preference was generally consistent with the respective patient's preferred choice for location of care, although no statistical comparison was performed due to the small sample size. The reasons given included "*With his condition, I wonder if [HITH] would have the medical equipment he needs*", "*for those times when he is especially bad*", and "*if he can be, for the convenience and comfort*". There was no association between caregiver satisfaction level and preferred site.

### Physicians' perspective

Fifteen attending physicians and 23 residents were involved in the care of study participants (seven family physicians and ten residents cared for 2 – 11 patients in the program). The patients had 31 different personal family physicians. Twelve attending physicians responded to at least one survey (27 responses). Those who did not respond were involved in a single admission. Only 9 residents completed surveys (13 responses), and 9 personal family physicians returned the survey. The most common reason for not responding was that they had no involvement in the specific care episode and had no information to provide.

**Figure 4:**
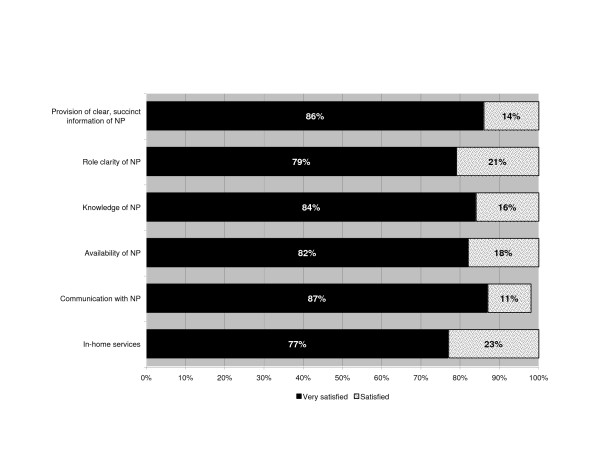
**Results of physician satisfaction survey**. Physician satisfaction with services.

**Figure 5:**
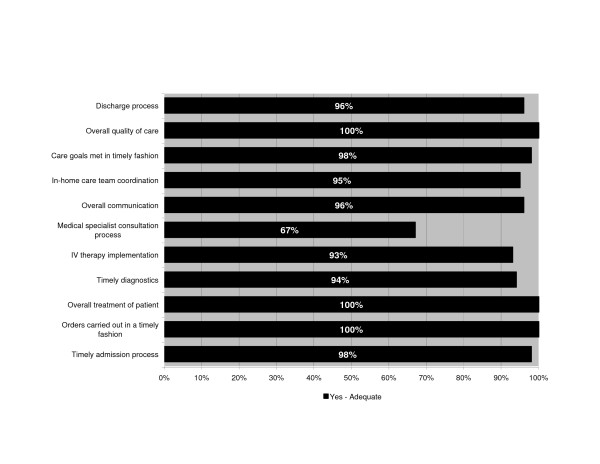
**Results of physician survey regarding program adequacy**. Physicians' perceptions of program adequacy.

Due to the small sample size and consistency of responses between the 3 groups (attendings, residents and family physicians), the results are presented combined. Virtually all respondents were very satisfied with the in-home services and the NP's performance (Figure 4). Eleven areas of the care process, such as timely admitting, timely diagnosis and overall quality of care were considered as adequate by 67% to 100% of physicians (Figure 5). Most admissions (66%) did not require a subsequent telephone call from the NP to the physician, and 30% required 1–3 calls (usually to the hospital resident). Some physicians (25%) reported having made 1–3 calls to the patient or their informal caregiver during the home hospitalization period. The median total estimated time spent on patient care by telephone by the physicians was 5 minutes per patient. When asked whether caring for the patients in the program affected their practice routine, the majority (88%) said "no" or "yes", but in a positive way. Of the remaining 12%, 3 said "yes" but did not provide further detail, one reported having to do a home visit, and two did not answer the question. Respondents were encouraged to provide general comments on the program. Of the 15 who provided written feedback none raised concerns. Comments were practical or positive such as "*Good program for this patient. Wife needs a lot of support*", "*Very good in concept but applicability is a bit limited. Would like to use that system more often*", and "*Allows us to follow patients without needing him to stay in hospital – excellent*".

### Practitioners' perspective

Six NPs provided care throughout the program, and 5 responded to the survey and participated in in-depth interviews. At the time of their interviews, 4 NPs were no longer actively working in the program. One NP, who could not be reached, had left the program after a very short stay. NPs who provided care had been practicing as nurse practitioners for an average of 5 years and had worked extensively in the field of nursing in diverse areas such as walk-in clinics, street outreach for homeless individuals, northern nursing, outpost nursing, palliative care, public health, and other forms of community nursing.

All NP respondents felt the patients received very good quality of care and that the program met (all or most of) the needs of their patients. NPs felt this was due to a combination of their advanced education; the amount of time the NPs were able to spend with each patient; and the information they provided on medications, chronic illness management, pain management, and caregiver support. "*I think the quality of care was exceptionally high... [W]e had the time to come to know individuals and their health care needs at a very intimate level*." NPs also highlighted some of the program drawbacks. They felt that clients in the HITH program did not have immediate access to diagnostic testing and specialists that would be available in a hospital environment. They also perceived that, while patients felt more control over their personal health needs, they experienced some day-to-day physical challenges such as having to prepare meals, and difficulty accessing bathroom facilities. They also voiced concerns over the continuity of care, when their involvement in patient care was limited to the few days of home hospitalization. **"***An ongoing problem in encounters with the health care system has been one of continuity of care. Patients are frequently expressing a sense of having been abandoned by a care provider that they have come to know." *Over the course of the program, NPs worked to address these concerns and improve this transition by collaborating on the development of a common care plan with the community home care agency.

There were challenges when developing relationships, defining roles and establishing program 'buy-in' with the medical staff, especially residents due to their bi-weekly rotations. " [*There was] little opportunity to develop relationships with residents. I am not sure there was enough "buy in" from docs; there was subtle resistance to it. That changed over time as more of the attendings came to know us. Even though they rotate every two weeks, eventually they get to know you*." However, NPs felt that the interdisciplinary team approach to care was beneficial to clients and important to establish.

The majority were satisfied with the extent of their participation in decision-making. They felt that they were given the opportunity to be autonomous and essentially drive the care of each patient. However, most NPs also felt that the NP profession was not the most appropriate for this position. While some said that the care did not require the expertise of an advanced practice nurse and that their skill set was not optimally utilized in the HITH study, others felt that patients were too ill to be cared for by NPs outside the hospital.

## Discussion

This study demonstrates that the provision of hospital level care in the home is acceptable to care providers and the majority of patients and their caregivers. Physicians felt the program offered excellent care and had minimal impact on physician workload. Nurse practitioners also rated the care very highly, but reported that the initial phase of the program required significant education of the medical staff, and that such a program would not exploit all of their skills. Patients and caregivers were highly satisfied with their care episode. However, a considerable number continued to have reservations about the safety of the program despite its very safe implementation. Four out of ten patients reported a preference for in-hospital admissions. This response possibly reflects apprehensions associated with exposure to a non traditional model of care, and one in which 24 hour on site coverage is not provided. Of the 44 admissions, only one patient required a home visit by the family physician. While this reflects non-problematic management of the patients, the absence of physician contact with patients may have impacted on patient perceptions of the program.

This pilot study has important limitations that require a full implementation study to address. We were unable to provide a contemporaneous comparison group, and the dimensions evaluated are subjective to the respondent's perspective and may be affected by a number of uncontrolled factors including setting, previous experience, knowledge, etc. The response rates to the follow-up surveys for all respondents were also mediocre, and we cannot rule out the possibility that the results, presented herein, are representative of a biased sample. Also, the study was set in an academic teaching practice, and the views expressed by the participants may not generalize to non university affiliated community based practices. Only 10% of individuals managed in the FMS ward during the study period were screened for participation in this study. The majority were felt to be too ill or too well at the onset to be considered. For a program of this type to be viable and to achieve economies of scale, it would need to be expanded to other hospital units. However, the limitations that we encountered in our pilot have been successfully addressed in large scale studies, so that successful implementation is feasible [6].

Consistent with two previous studies, patients and their informal caregivers reported high levels of satisfaction with the care received through this hospital replacement program [2,14]. In many instances, patients and families were found to enjoy comfort and independence when cared for in the home [14-17] a factor that corresponds with the values for change within the Canadian health care system [1] (*pp. 171–172*). The current study supports this finding through the perspective of both patients and informal caregivers. In randomized controlled trials, the satisfaction level of patients treated in the home was at least comparable with those treated in hospital [18].

Informal caregivers were mostly females, and not working for pay. Despite the novelty of the program, virtually all were comfortable with their role as the caregiver. Other studies have shown that informal caregivers are confident in their role; that they would act as caregivers again; and they would recommend the role to others [19]. In the current study, some informal caregivers reported household costs associated with treatment through the program. Randomized controlled studies suggest that costs to the family are considerably lower for patients treated at home than for in-patients [20]. Caregivers of hospitalized patients must contend with transportation costs, parking, meals outside the house and other expenses.

Despite a high level of satisfaction, approximately one third of patients and informal caregivers expressed a preference for hospital over home for their care. These were more often observed in patients with more complex illnesses, although this did not reach statistical significance. The most commonly cited reason for this choice was concern about the availability of emergency services should the need arise. This may reflect the novelty of the program and a public's lack of awareness of the extent to which such programs are safely implemented in other regions. Supporting participants with additional information, at the time of admission, may improve comfort levels with the program. Randomized controlled trials showed that the level of burden on informal caregivers, of patients managed in the home, was not different from those managed in hospital and that the disruption was lower [15,21]. However, our results support Shepperd and Ilife's recommendation that the views of the carers be taken into account [18,22] in the decision to admit to HITH [21].

The NPs offered comprehensive levels of care and provided the patients with information concerning the nature of their illness along with functional measures to better self-manage their health. These were key features in the success of other studies [14,16,17]. Provider perspectives of HITH have not been as closely examined as patient and informal caregiver perspectives in the existing literature. Nonetheless the current results agree with previous studies, which indicate that providers feel these programs deliver quality care to the patient [23]. NPs were satisfied that the program met patient needs but were not as satisfied with their own role in the program. It is possible that the NPs could not exploit several aspects of the additional Primary Care NP nursing education program they had received in this acute hospital level setting. The province of Ontario is now training Extended Class Nurses with additional training for hospital patients. Future HITH interventions should pay close attention to the definition of respective roles of clinicians within the program to ensure skills are effectively applied to the care process. Other countries with widely implemented HITH programs generally rely on nurses who specialize in the condition being managed or on experienced nurses for the management of general conditions. This approach would likely be appropriate in Canada as well.

Concern about the increase in workload to general practitioners associated with HITH was not reflected in the current study [8]. The physicians indicated their support for the program and found that it had a positive impact on their practice and took a minimal amount of their time. While a good representation of hospital attending physicians was reflected in the responses, residents and community physicians were less responsive. None-the-less the study suggests the program is acceptable to family practitioners practicing in the hospital and community setting.

This study builds on Canada's experience in the use of home hospitalization as a viable alternative to in-hospital care. While few reports are published on these Canadian programs, those available support their use [24-26].

## Conclusion

Patients were recruited from an academic hospital in an urban centre. The efficacy (health benefit) of HITH programs will depend on a number of factors in the health-care environment and also on the selection of appropriate patients for the program. In our setting the care processes involved in the HITH program were acceptable to patients, informal caregivers and healthcare providers. The impact of the program on the physician's workload was minimal, but the NPs, educated as Primary Care Nurse Practitioners, were not satisfied with their role in the program. The role of a 'Hospital in the Home' Nurse Practitioner requires further investigation as a longer term strategy to deal with an expansion of hospital services that can be delivered at home. During program implementation, attention should be paid to educating participants about the safety of such programs and clarifying the functional roles of clinicians. The results suggest that patients and informal caregivers are prepared to accept this intervention as a positive change that will help the future health system better align resources to needs.

Encouraging findings from our pilot program and contemporary literature support further development of this model of Hospital in the Home into a fully fledged program.

## Competing interests

The author(s) declare that they have no competing interests.

## Authors' contributions

The authors contributed to the article as follows: WEH, JL, RS and GV conceived the hospital in the home program. WEH and JL managed the implementation of program and lead the study. Clinical management meetings: JL and WEH, CDA, SD, JAD and JPN assisted with ongoing conception and design, drafting of data collection instruments and other materials and assisted with data analysis/interpretation. WZ assisted with the drafting of data collection instruments and other materials as well as data analysis and interpretation. All authors have given approval to the manuscript presented here.

## Pre-publication history

The pre-publication history for this paper can be accessed here:


